# Norepinephrine transporter variant A457P knock-in mice display key features of human postural orthostatic tachycardia syndrome

**DOI:** 10.1242/dmm.012203

**Published:** 2013-04-04

**Authors:** Jana K. Shirey-Rice, Rebecca Klar, Hugh M. Fentress, Sarah N. Redmon, Tiffany R. Sabb, Jessica J. Krueger, Nathan M. Wallace, Martin Appalsamy, Charlene Finney, Suzanna Lonce, André Diedrich, Maureen K. Hahn

**Affiliations:** 1 Division of Genetic Medicine, Vanderbilt University School of Medicine, Nashville, TN 37232, USA; 2 Department of Medicine, Vanderbilt University School of Medicine, Nashville, TN 37232, USA; 3 Department of Pharmacology, Vanderbilt University School of Medicine, Nashville, TN 37232, USA; 4 Vanderbilt Kennedy Center for Research on Human Development, Vanderbilt University School of Medicine, Nashville, TN 37232, USA

## Abstract

Postural orthostatic tachycardia syndrome (POTS) is a common autonomic disorder of largely unknown etiology that presents with sustained tachycardia on standing, syncope and elevated norepinephrine spillover. Some individuals with POTS experience anxiety, depression and cognitive dysfunction. Previously, we identified a mutation, A457P, in the norepinephrine (NE; also known as noradrenaline) transporter (NET; encoded by *SLC6A2*) in POTS patients. NET is expressed at presynaptic sites in NE neurons and plays a crucial role in regulating NE signaling and homeostasis through NE reuptake into noradrenergic nerve terminals. Our *in vitro* studies demonstrate that A457P reduces both NET surface trafficking and NE transport and exerts a dominant-negative impact on wild-type NET proteins. Here we report the generation and characterization of NET A457P mice, demonstrating the ability of A457P to drive the POTS phenotype and behaviors that are consistent with reported comorbidities. Mice carrying one A457P allele (*NET*^+/P^) exhibited reduced brain and sympathetic NE transport levels compared with wild-type (*NET^+/+^*) mice, whereas transport activity in mice carrying two A457P alleles (*NET*^P/P^) was nearly abolished. *NET*^+/P^ and *NET*^P/P^ mice exhibited elevations in plasma and urine NE levels, reduced 3,4-dihydroxyphenylglycol (DHPG), and reduced DHPG:NE ratios, consistent with a decrease in sympathetic nerve terminal NE reuptake. Radiotelemetry in unanesthetized mice revealed tachycardia in *NET*^+/P^ mice without a change in blood pressure or baroreceptor sensitivity, consistent with studies of human NET A457P carriers. *NET*^+/P^ mice also demonstrated behavioral changes consistent with CNS NET dysfunction. Our findings support that NET dysfunction is sufficient to produce a POTS phenotype and introduces the first genetic model suitable for more detailed mechanistic studies of the disorder and its comorbidities.

## INTRODUCTION

Norepinephrine (NE; also known as noradrenaline) is the primary signaling neurotransmitter of the autonomic sympathetic nervous system (SNS), where it acts to control heart rate (HR). Compromised NE signaling has been associated with autonomic disease, particularly impacting cardiovascular function (Billman et al., 1997; Esler and Kaye, 2000; Exner et al., 1999; Parati and Esler, 2012). NE signaling is also implicated in disorders of anxiety, depression and cognition (Beane and Marrocco, 2004; Pliszka, 2005; Ressler and Nemeroff, 1999). Released NE is inactivated through active transport into noradrenergic terminals by the presynaptically localized Na^+^/Cl^–^-dependent NE transporter (NET; encoded by *SLC6A2*; note that throughout this paper the gene is referred to as *NET*) (Iversen, 1963; Matthies et al., 2009). NET is responsible for the reuptake of as much as 90% of released NE and thus plays a crucial role in NE inactivation and homeostasis (Schömig et al., 1989; Xu et al., 2000). Altered sympathetic nerve terminal NET uptake sites and/or activity are found in hypertension, diabetes, cardiomyopathy, heart failure and ischemia (Böhm et al., 1998; Merlet et al., 1992; Rumantir et al., 2000; Schnell et al., 1996; Schömig et al., 1991).

We identified a link between a NET genetic mutation with a familial incidence of postural orthostatic tachycardia syndrome (POTS) (Shannon et al., 2000; Hahn et al., 2003). POTS is characterized by an increase in standing heart rate that is not accompanied by hypotension (Robertson, 1999). Idiopathic POTS is accompanied by features that suggest a failure of NET, including increased NE spillover, and decreased NE clearance, intraneuronal NE metabolism and response to tyramine (Furlan et al., 1998; Jacob et al., 1999). In normal human subjects, pharmacological NET blockade produces orthostatic tachycardia much like that observed in POTS (Mayer et al., 2006; Schroeder et al., 2002). Some POTS patients experience anxiety, depression and cognitive dysfunction (Raj et al., 2009). In a search for a molecular basis for the POTS diagnoses of twin probands, Shannon et al. identified a nonsynonymous mutation in the *NET* gene that produces the amino acid substitution A457P in transmembrane domain 9 of the NET protein (Shannon et al., 2000). In a within-family analysis, A457P carriers demonstrated standing-induced increased HR and plasma NE, and a decreased plasma 3,4-dihydroxyphenylglycol (DHPG):NE ratio, relative to family members not carrying the mutation (Shannon et al., 2000). Subsequent *in vitro* expression studies revealed that A457P disrupts normal plasma membrane targeting, and leads to a severe loss of NE transport activity (Hahn et al., 2003; Shannon et al., 2000). A457P acts dominantly *in vitro* to disrupt the trafficking and transport activity of wild-type NET protein, consistent with the presence of POTS in A457P carriers, who are heterozygotes. Recently, Lambert and colleagues reported reduced levels of NET protein in sympathetic nerve biopsies of POTS patients (Lambert et al., 2008), suggesting that NET dysfunction might be a common feature of the disorder.

**TRANSLATIONAL IMPACT**

**Clinical issue**

Postural tachycardia syndrome (POTS) is a condition of autonomic nervous system dysfunction that is characterized by persistent tachycardia when moving from a supine to an upright position and, in many patients, elevation of norepinephrine (NE). This surge in NE levels has been predicted to drive tachycardia. POTS can be severely debilitating, with patients generally experiencing a poor heath-related quality of life, comparable to patients with chronic obstructive pulmonary disease and congestive heart failure. Despite the known involvement of the autonomous nervous system, the molecular mechanisms and genetics underlying POTS remain largely unknown. Biochemical and physiological evidence points to impairment in the norepinephrine transporter (NET) as one causal factor. In normal circumstances, NET plays a crucial role in regulating NE signaling and homeostasis in both the brain and sympathetic nervous system by clearing NE at noradrenergic nerve terminals. It is hypothesized that defects in reuptake would result in excess NE at sympathetic nerve terminals, giving rise to the POTS phenotype. Owing to its role in the CNS, the transporter has also been implicated in the pathophysiology of several mental health disorders.

**Results**

Previously, this group identified and characterized the first genetic contributor to POTS, a variant in the *NET* gene, by investigating familial cases. In the current study, they generate and characterize mouse models expressing the variant (NET A457P) to establish an animal model of POTS. They report that NET A457P knock-in mice have the POTS phenotype as well as behaviors consistent with reported co-morbidities. Heterozygote mice carrying one A457P allele (*NET*^+/P^) exhibited reduced brain and sympathetic NE transport levels compared with wild-type mice (*NET*^+/+^), whereas transport activity in homozygotes (*NET*^P/P^) was virtually abolished. In addition, the knock-in mice exhibited elevations in plasma and urine NE levels, reduced 3,4-dihydroxyphenylglycol (DHPG), and reduced DHPG:NE ratios, consistent with a decrease in sympathetic nerve terminal NE reuptake. The group performed radiotelemetry in unanesthetized mice, which revealed tachycardia in heterozygotes without a change in blood pressure or baroreceptor sensitivity, consistent with studies of human NET A457P carriers. Finally, *NET*^+/P^ mice are reported to demonstrate anxiety behaviors that are consistent with CNS NET dysfunction.

**Implications and future directions**

These findings confirm that NET A457P is sufficient to produce the POTS phenotype, and further corroborate the link between POTS and NET activity. Although most individuals with POTS do not carry the A457P mutation, it is important to note that NET impairment has been reported even in such cases. Inter-individual variation in human NET activity is likely to be common and clinically important in a range of disorders, in light of the transporter’s central role in NE regulation. Thus, NET A457P mice could have broad importance and utility as a model for detailed mechanistic studies of POTS and its comorbidities. Understanding how NET contributes to both cardiovascular disease and mental health disorders could have implications for the development of novel treatment strategies.

In the current study, we generated NET A457P knock-in mice to establish an animal model of POTS with construct validity. Below, we describe features of the A457P knock-in mouse that lead us to conclude that the NET A457P substitution is sufficient to produce the key features of POTS as well as its comorbidities.

## RESULTS

**Fig. 1.**
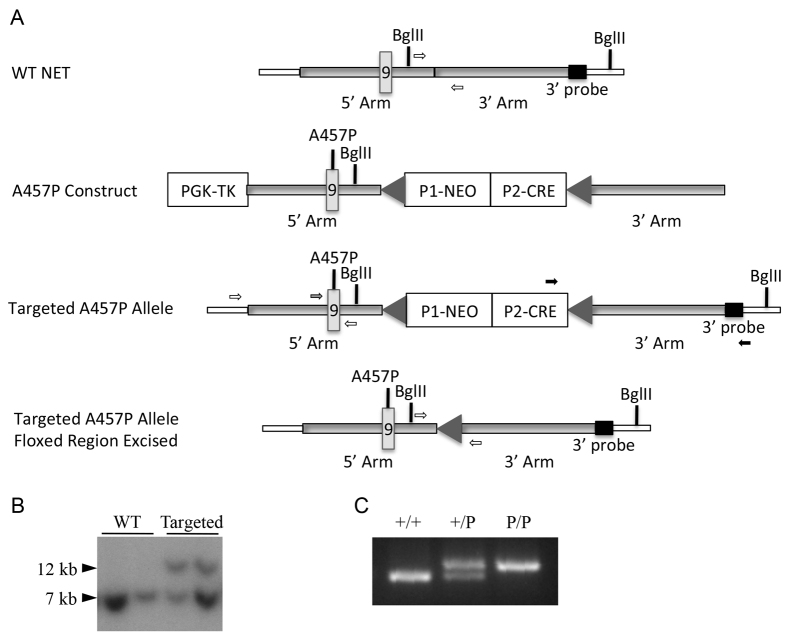
**Generation of NET A457P knock-in mice.** (A) Schematic of the wild-type (WT) *SLC6A2* (*NET*) locus, targeting vector and NET locus following recombination. ‘WT NET’ shows the location of the targeting arms, and *Bgl*II sites and 3′ probe used in Southern confirmation. White arrows depict the PCR primers used to genotype A457P mice. ‘A457P Construct’ depicts the position of the floxed Neo/CRE cassette and the PGK cassette. Neo is under the control of the RNA polymerase II promoter and CRE is driven by the testis-specific angiotensin converting enzyme promoter, producing excision of the floxed region upon germline targeting. A457P in exon 9 in the 5′ arm is depicted. ‘Targeted A457P Allele’ illustrates the construct targeted to the *NET* locus. The *Bgl*II sites and 3′ probe location illustrate the shift in size of the *Bgl*II fragment, produced by insertion of the construct, to 11.6 kb (from 7.8 kb in WT NET). Arrows indicate PCR primers used to confirm correct insertion at both the 5′ (white arrows) and 3′ (black arrows) end of *NET*. The gray arrow shows a forward primer used with the 5′ arm reverse primer (white arrow) to genotype the A457P mutation through PCR and dideoxy sequencing. ‘Targeted A457P Allele Floxed Region Excised’ shows the final status of the *NET* allele, with the Neo/CRE cassette excised and one *loxP* site remaining in intron 11. White arrows depict the PCR primers used to genotype A457P mice, as shown above in WT NET. (B) Southern analysis of *Bgl*II-digested DNA from *NET*^+/+^ and *NET*^+/P^ mice. (C) PCR genotype analysis of *NET*^+/+^, *NET*^+/P^ and *NET*^P/P^ mouse DNA to detect the residual *loxP* site.

*NET* A457P knock-in mice were generated through a homologous recombination strategy that introduced A457P into exon 9 of the mouse gene at the position orthologous to the identified human A457P mutation (Fig. 1A). Embryonic stem (ES) cell clones were confirmed for correct homologous recombination of both the 5′ and 3′ arms through Southern blot (Fig. 1B) and PCR. The presence of A457P was also determined through dideoxy sequencing. Heterozygous (*NET*^+/P^) and homozygous (*NET*^P/P^) A457P mice were identified by PCR genotyping strategy to detect a residual *loxP* site in intron 11 (Fig. 1C), and by sequencing of the A457P mutation. A ‘speed congenics’ breeding strategy was used to backcross mice to the C57BL/6 strain (Jackson Laboratories, Bar Harbor, ME) (Wong, 2002). Mice appeared healthy, bred with expected genotype distributions and exhibited no differences in body weight among genotypes for either male or female mice (data not shown).

**Fig. 2.**
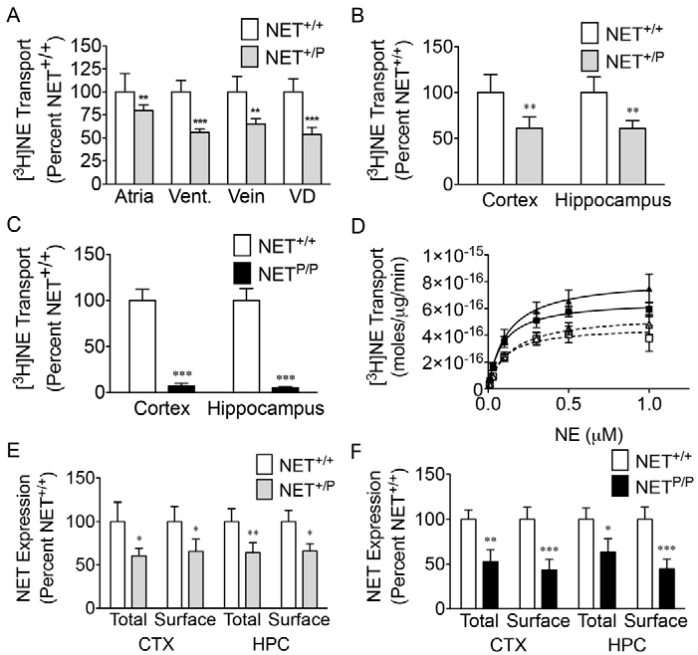
**[^3^ H]NE transport and expression in A457P knock-in mice.** Data are expressed as mean percent ± s.e.m. of *NET*^+/+^ mice (A–C,E,F) or as raw values ± s.e.m. (D). *P<0.05, **P<0.01 and ***P<0.001 compared with *NET*^+/+^ mice (two-tailed *t*-test). (A) [^3^H]NE transport in the SNS, *n*=5–6. Vent., ventricle; VD, vas deferens. (B,C) Single point [^3^H]NE transport in brain, *n*=6–7. (D) Saturation kinetics of [^3^H]NE transport in brain, *n*=8. Cortex *NET*^+/+^ (black squares), *V*_MAX_=6.6±0.40 moles×10^−16^/μg/minute, *K*_M_=87.8±20.9 nM; cortex *NET*^+/P^ (white squares), *V*_MAX_=4.6±0.50 moles×10^−16^/μg/minute, *K*_M_=96.4±38.1 nM; hippocampus *NET*^+/+^ (black triangles), *V*_MAX_=8.2±0.77 moles×10^−16^/μg/minute, *K*_M_=118.3±40.6 nM; hippocampus *NET*^+/P^ (white triangles), *V*_MAX_=5.5±0.54 moles×10^−16^/μg/minute, *K*_M_=136.8±46.3 nM. (E,F) NET expression in total and surface fractions of cortex (CTX) and hippocampus (HPC) synaptosomes in *NET*^+/P^ (*n*=7; E) and *NET*^P/P^ (*n*=7; F) mice compared with *NET*^+/+^ mice.

*NET*^+/+^, *NET*^+/P^ and *NET*^P/P^ mice were assessed for NET expression and activity to determine whether the deficits observed for A457P under heterologous expression would translate to noradrenergic neurons. Initial experiments determined similar changes in male and female A457P mice, and thus western blot and single point transport data from male and female A457P mice were combined for analysis. In assays assessing the *ex vivo* transport of radiolabeled NE in peripheral noradrenergic terminals, *NET*^+/P^ mice demonstrated significant transport decreases in the atria and ventricle of the heart, vas deferens and femoral vein (Fig. 2A; *n*=5–6). This is supportive of the hypothesis that a deficit in NET activity in the SNS, particularly in the heart, drives the phenotype in heterozygous human A457P carriers.

We next sought to determine whether these transport deficits extended to CNS noradrenergic neurons. In synaptosomes prepared from cortex and hippocampus, transport levels in *NET*^+/P^ mice were ∼60% of *NET*^+/+^ mouse levels. Saturation kinetic experiments in cortical and hippocampal synaptosomes from male *NET*^+/P^ mice demonstrated a decrease in maximal velocity of reaction (*V*_MAX_) values, with no change in the substrate concentration required for 50% V_MAX_ (*K*_M_) for NE (Fig. 2D; *P*<0.05, *n*=8). In *NET*^P/P^ mice, transport was nearly abolished (Fig. 2C). Thus, the A457P protein exhibits very little if any transport *in vivo*, as we previously observed *in vitro* (Hahn et al., 2003).

NET expression was determined in total and surface fractions of synaptosomal preparations using a membrane–impermeant biotinylation assay. *NET*^+/P^ and *NET*^P/P^ mice demonstrated decreased NET expression in both the cortex and hippocampus (Fig. 2E,F). *NET*^+/P^ mice demonstrated ∼60% of *NET*^+/+^ total NET levels, and displayed similar decreases in surface NET pools (Fig. 2E). *NET*^P/P^ demonstrated loss of NET protein in cortical and hippocampal terminals similar to that of the *NET*^+/P^ mice (Fig. 2F). Thus, unlike a *NET* gene deletion, A457P mice retain a high level of protein expression of a nonfunctional transporter that might play a role in influencing normal NET function or trafficking in *NET*^+/P^ mice. Overall, these results are consistent with the pattern of loss of A457P expression and plasma membrane trafficking previously demonstrated using transfected cells.

**Fig. 3.**
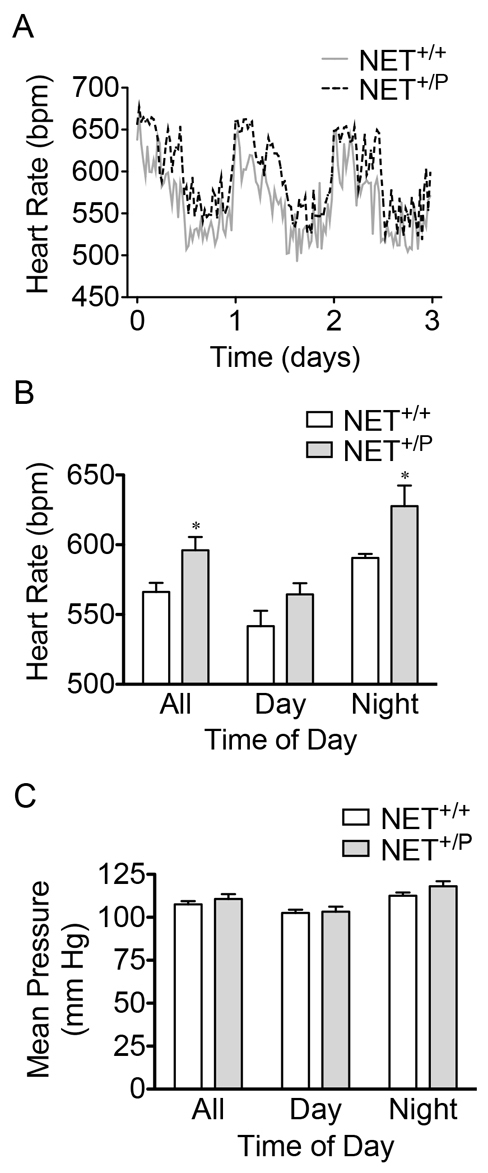
**Radiotelemetry in *NET*^+/+^ versus *NET*^+/P^ mice.** (A) 3-day collection of HR measures; data were analyzed and graphed in 30-minute increments. Traces shown start at lights off in the colony room. (B) HR during 3 days of data collection, expressed as means ± s.e.m. averaged over the 24-hour period and during 12-hour day and night periods. Significance was determined by twoway ANOVA. Genotype: *F*(1,27)=15.59, *P*<0.001. Time of day: *F*(2,27)=18.26, *P*<0.0001. *P<0.05, post-hoc *t*-test, *n*=5. (C) Mean arterial BP measured during the same 3-day period as in A and B (*n*=5).

**Fig. 4.**
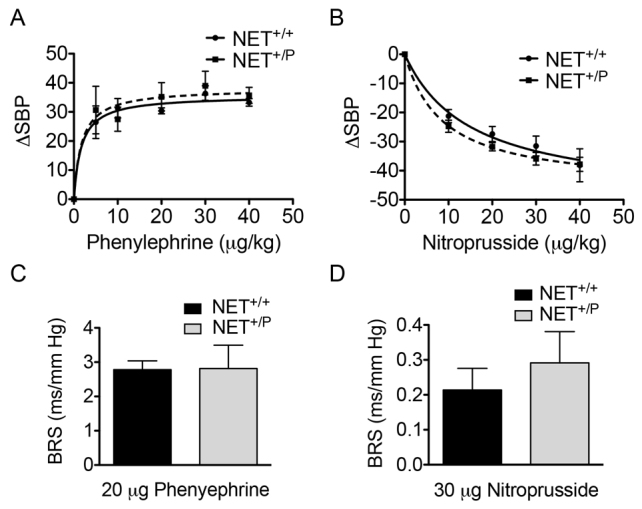
**Baroreflex sensitivity in *NET*^+/+^ versus *NET*^+/P^ mice.** (A,B) Dose response of systolic BP (SBP) to PHE (A) and NTP (B). Two-way ANOVA revealed effects of dose for both PHE and NTP [*F*(4,50)=61.78, *P*<0.01, *n*=3–6 for NTP and *F*(5,60)=23.53, *P*<0.01, *n*=3–6 for PHE]. (C,D) Baroreceptor sensitivities [the ratio of the change in HR to the changes in SBP at 20 μg/kg PHE (C) and 30 μg/kg NTP (D)] were calculated. BRS, baroreflex sensitivity; PHE, phenylephrine; NTP, nitroprusside.

We next assessed whether the NET deficit altered cardiovascular function in *NET*^+/P^ mice similar to that of human A457P carriers. Because A457P carriers are heterozygotes, it was crucial to determine whether *NET*^+/P^ mice could drive the cardiovascular phenotypes. Indeed, we demonstrated that the presence of one A457P allele in *NET*^+/P^ mice is sufficient to produce deficits in NET expression and uptake. Here, we ascertained cardiovascular phenotypes in *NET*^+/P^ mice, using radiotelemetry to monitor blood pressure (BP) and HR in conscious, freely behaving mice. Fig. 3A depicts HR over the course of 3 days of data collection. HR of both *NET*^+/+^ and *NET*^+/P^ mice followed a diurnal rhythm with lower and higher HRs during rest (during the daytime/lights on in the colony room) and active (night time/lights off) periods, respectively. Two-way ANOVA revealed a significant effect of both genotype and time of day on HR [*F*(1,27)=15.59, *P*<0.001 for genotype and *F*(2,27)=18.26 for time of day]. *NET*^+/P^ mice exhibited higher HR over both the entire 24-hour period and at night (Fig. 3B; *P*<0.05, *n*=5). A457P carriers, and other individuals with POTS that also exhibit excessive plasma NE, do not exhibit the hypotension that accompanies other forms of orthostatic intolerance (Shannon et al., 2000). In this group of patients, BP remains unchanged or becomes somewhat hypertensive during standing. Mean arterial BP, measured during the same recording period as HR, was unchanged in *NET*^+/P^ compared with *NET*^+/+^ mice (Fig. 3C). Neither diastolic nor systolic BPs were different between genotypes (data not shown). We also determined whether there was a change in the baroreflex sensitivity in A457P knock-in mice. Here, we measured baroreceptor-mediated changes in the HR response following intravenous administration of phenylephrine (PHE) or nitroprusside (NTP) in anesthetized *NET*^+/+^ or *NET*^+/P^ mice. Twoway ANOVA revealed effects of dose for both PHE and NTP [*F*(4,50)=61.78, *P*<0.01, *n*=3–6 for NTP and *F*(5,60)=23.53, *P*<0.01, *n*=3–6 for PHE] (Fig. 4A,B). However, there were no differences in the BP response between *NET*^+/+^ and *NET*^+/P^ mice following PHE or NTP administration. Baroreceptor sensitivity (BRS), the ratio of the change in HR to the change in BP at an effective dose of both PHE and NTP, was not different between genotypes (Fig. 4C,D; *n*=6; PHE, 20 μg/kg body weight; NTP, 30 μg/kg body weight).

**Fig. 5.**
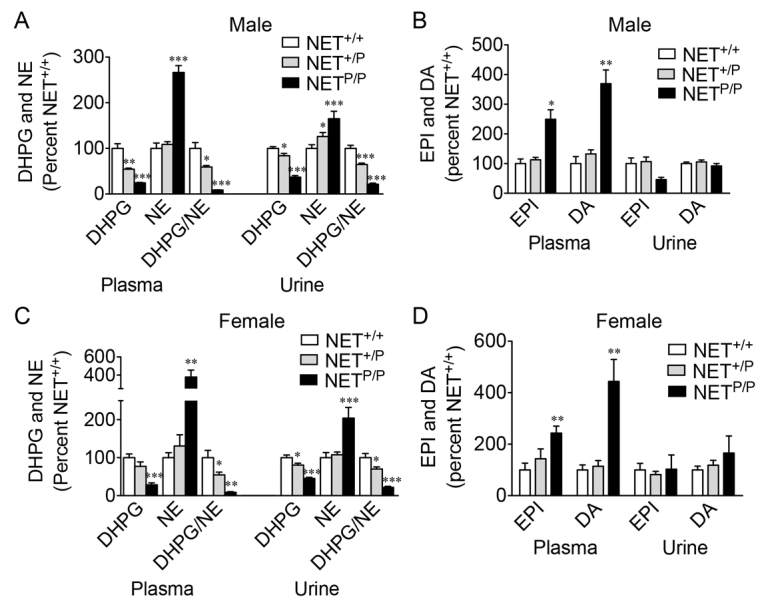
**Catecholamines and metabolites in A457P knock-in mice.** Plasma and urinary NE and DHPG (A,C), and DA and EPI (B,D) in male (A,B) and female (C,D) A457P mice. Data are expressed as the mean percent ± s.e.m. compared with *NET*^+/+^ mice. Significance was determined by two-way ANOVA. Male plasma genotype, *F*(2,135)=50.77, *P*<0.001; male urine genotype, *F*(2,215)=10.82; *P*<0.001; female plasma genotype, *F*(2,110)=20.21, *P*<0.001; female urine interaction, *F*(8,85)=2.88, *P*<0.01. *P<0.05, **P<0.01 and ***P<0.001 compared with *NET*^+/+^. *n*=6–12 (male plasma), 9–20 (male urine), 8–9 (female plasma) and 6–7 (male urine).

One of the defining characteristics that we attribute to NET deficiency in A457P carriers that is also a salient feature in other POTS patients, is a disturbance in the homeostasis of peripheral NE indicated by changes in the DHPG:NE ratio. We attribute this to a lack of NE reuptake that results in both elevated NE in plasma and urine and a decrease in the intraneuronal metabolism of NE to DHPG. Other individuals with POTS, although they do not harbor the A457P mutation, have also been hypothesized to have faulty NE uptake as part of the disease phenotype. Indeed, recent studies support loss of NET protein in other POTS patients (Lambert et al., 2008). We sought to determine whether the introduction of A457P into mice was sufficient to recapitulate this phenotype. Plasma and urinary NE and DHPG were measured in mice under resting conditions. Two-way ANOVA revealed significant effects in both male and female mice [male plasma genotype, *F*(2,135)=50.77, *P*<0.001, *n*=6–12; male urine genotype, *F*(2,215)=10.82; *P*<0.001, *n*=9–20; female plasma genotype, *F*(2,110)=20.21, *P*<0.001, *n*=8–9; female urine interaction, *F*(8,85)=2.88, *P*<0.01, *n*=6–7). NE levels were elevated, DHPG levels were decreased and the DHPG:NE ratio was decreased, highly consistent with those abnormalities observed in human A457P carriers (Fig. 5A,C, *P*<0.05-0.01). These effects were observed in *NET*^+/P^ mice and *NET*^P/P^ mice, consistent with the ability of a single copy of the mutant allele, as seen in the POTS patients where it was identified, to confer these changes in NE homeostasis. In some cases, NE increases were not significant in *NET*^+/P^ mice and this might be attributable to the collection of both plasma and urine under anesthetized and non-stressful conditions, respectively. Indeed, family members with A457P exhibit elevated standing plasma NE levels, whereas supine NE levels are much less affected. Dopamine (DA) and epinephrine (EPI; also known as adrenaline) levels were also increased in male and female *NET*^P/P^ mice in plasma but not urine (Fig. 5B,D; *P*<0.05-0.01).

We also investigated whether impaired NE reuptake influences tissue levels of catecholamines and metabolites. There was a trend for female *NET*^P/P^ mice to have smaller hearts compared with wild-type females (data not shown; *P*=0.08). DHPG levels were decreased in heart of *NET*^+/P^ and *NET*^P/P^ mice, and NE levels and the DHPG:NE ratio were decreased in *NET*^P/P^ mice (Table 1). There was a trend for a decrease in the DHPG:NE ratio in male *NET*^+/P^ heart (*P*=0.08). EPI levels were also decreased in hearts of A457P knock-in mice, with a 30% decrease in *NET*^+/P^ (*P*=0.09) mice and a significant 60% decrease in *NET*^P/P^ mice. Tissue DA levels were unchanged.

**Table 1.**
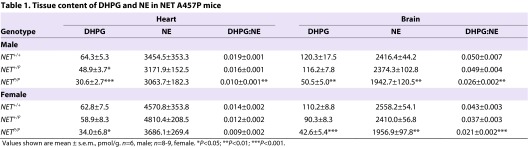
Tissue content of DHPG and NE in NET A457P mice

**Fig. 6.**
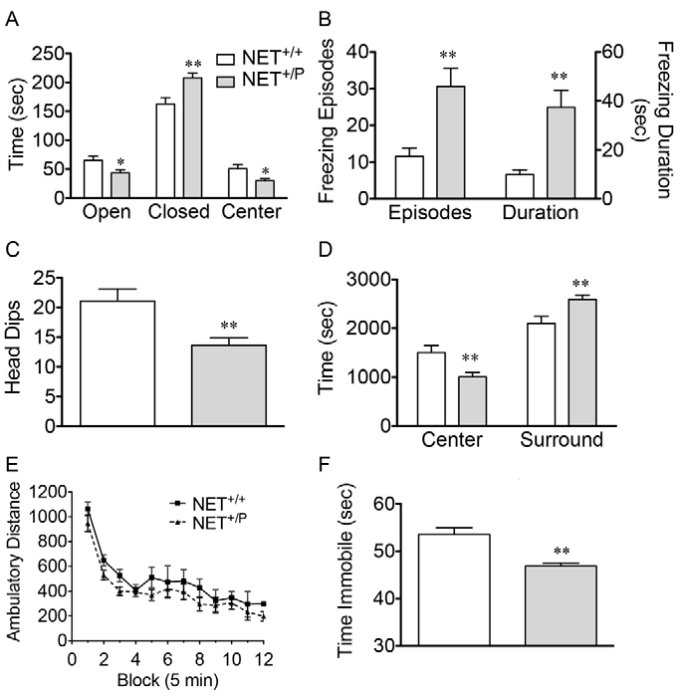
***NET*^+/+^ versus *NET*^+/P^ mice in behavioral tests.** Data are expressed as mean ± s.e.m. (A–C) Behavior in the EPM. For time spent in the different zones (A), significance was determined by two-way ANOVA. Interaction: *F*(2,96)=13.74, *P*<0.0001. *n*=16–18. Freezing behavior (B) and head dips (C) were also assessed. *n*=16–18. (D,E) Open field exploration. Time spent in the center versus surrounding zones (D) and distance travelled (E) were measured. *n*=16–18. (F) Time immobile in the last 4 minutes of the TST. *n*=7–9. *P<0.05, **P<0.01, post-hoc *t*-test.

We also investigated the impact of impaired NE reuptake to produce dysfunction in the CNS. There were no differences between *NET*^+/+^ mice and either *NET*^+/P^ or *NET*^P/P^ mice in brain weights (data not shown). Consistent with the transport activity deficits observed, NE and DHPG levels and the DHPG:NE ratio were decreased in the brain of male and female *NET*^P/P^ mice, although there were no significant changes in *NET*^+/P^ mice (Table 1). The role of NET in anxiety and depression, and the presence of these symptoms in some POTS subjects, led us to explore whether A457P mice might exhibit phenotypes related to these disorders. Because A457P carriers are heterozygotes and *NET*^+/P^ mice showed deficits in CNS NET expression and NE uptake, we focused behavioral tests on *NET*^+/P^ mice. First, mice were exposed to the elevated plus maze (EPM), which measures an animal’s anxiety based on the conflict between the motivation to approach and explore novel situations versus a fear of open, brightly lit spaces (open arms). Additionally, freezing and head dips, ethological measures of fear- and anxiety-related behaviors sensitive to anxiolytic compounds, were assessed in the EPM (Blanchard et al., 2003; Gerits et al., 2007; Gross et al., 2000; Rosen and Schulkin, 1998; Wall et al., 2004; Weiss et al., 1998). We detected a highly significant interaction effect in the EPM between genotype and compartment [*F*(2,96)=13.74, *P*<0.0001]. *NET*^+/P^ mice spent less time in the open arms and more time in the closed arms of the maze compared with *NET*^+/+^ mice (Fig. 6A; *P*<0.05 and 0.01, respectively, *n*=16–18). Additionally, *NET*^+/P^ mice exhibited a significant increase in freezing incidence as well as durations of freezing as compared with *NET*^+/+^ mice and also exhibited fewer head dips, all of which are behaviors that are indicative of anxiety (Fig. 6B,C; *P*<0.01, *n*=16–18). We detected no differences in the number of entries into any of the zones, indicating a lack of alteration in general motor activity level (data not shown) (Braun et al., 2011). Anxiety-like behavior was also detected in the open field, where *NET*^+/P^ mice spent significantly less time in the center of the field and increased time along the periphery (thigmotaxis) (Fig. 6D; *P*<0.01, *n*=16–17). As with indications of normal general activity in the EPM, *NET*^+/P^ mice exhibited no difference in total distance travelled in the open field (Fig. 6E). Finally, compared with *NET*^+/+^ mice, *NET*^+/P^ mice exhibited decreased immobility in the tail suspension test (TST), a test that is sensitive to NE-enhancing antidepressant medication (Fig. 6F; *P*<0.05, *n*=7–9).

## DISCUSSION

*NET*^+/P^ mice exhibited elevated HRs, supporting our prior contention based on within-family measure of phenotype associations that a single copy of the A457P allele is responsible for the tachycardia that is evident in human heterozygous A457P carriers. We suspect that the larger increase in HR during the active period reflects increased sympathetic activity during this time. Greater NE release during the active period probably renders synaptic NE levels, NE spillover and HR more dependent on NE reuptake. Interestingly, BP was not affected by the A457P allele, similar to the phenotype observed in human A457P carriers and most idiopathic POTS subjects (Shannon et al., 2000). This finding could be explained by the fact that NET removes greater amounts of NE released in heart compared with other sympathetically innervated organs (Goldstein et al., 1988). We did not observe a change in baroreflex sensitivity. Some studies have reported altered baroreflex sensitivity in POTS patients whereas others have found no differences (Farquhar et al., 2000; Furlan et al., 1998; Jacob et al., 1999; Jordan et al., 2002; Muenter Swift et al., 2005). Regardless, our results indicate that the tachycardia induced by A457P does not seem to be directly mediated by changes in baroreflex sensitivity.

Previously, we demonstrated both trafficking and transport deficits of A457P *in vitro*, with a decrease in surface NET expression and a more severe, near total, loss of transport activity (Hahn et al., 2003). Consistent with these studies, A457P mice are deficient in both NET surface expression and NE transport activity in native SNS and CNS noradrenergic nerve terminals, with *NET*^P/P^ mice exhibiting near total loss of transport. Previous *in vitro* studies also demonstrated that NET A457P physically associates with wild-type NET transporters and hinders them from reaching the cell surface, serving to decrease wild-type NET transport activity (Hahn et al., 2003). Evidence from other studies supports that neurotransmitter transporters form oligomer complexes early in the secretory pathway that are maintained in the plasma membrane, suggesting the likelihood of both physical and functional interactions among wild-type and mutant transporter species (Bartholomäus et al., 2008; Hahn et al., 2003; Hastrup et al., 2001; Kilic and Rudnick, 2000; Scholze et al., 2002; Sitte et al., 2004; Torres et al., 2003). Data presented here suggest that a dominant negative effect of A457P might contribute, in part, to deficits *in vivo* in *NET*^+/P^ mice. First, observations from our laboratory indicate that heterozygous *NET* knockout mice do not express significant changes in NE transport (data not shown), whereas *NET*^+/P^ mice exhibited significant transport deficits in the present study. Additionally, changes in DHPG levels and DHPG:NE ratios were altered in plasma and urine in *NET*^+/P^ mice in our study, whereas no changes were detected in DHPG levels in the heterozygous NET knockout mouse (Keller et al., 2004). The consequences of expression of an impaired NET protein in NE neurons might additionally impact the interaction of NET with its multiple protein partners (Bauman et al., 2000; Carneiro et al., 2002; Sung et al., 2003; Torres et al., 2001; Wersinger et al., 2006).

The loss of NE reuptake due to A457P was demonstrated as increased NE and decreased intraneuronal metabolism of NE (both decreased DHPG levels and decreased DHPG to NE ratio) in the periphery, consistent with the human phenotype. In some cases, NE increases were not significant in the *NET*^+/P^ mice. Consistent with these findings, A457P family members exhibit highly elevated standing plasma NE levels, whereas supine NE levels are much less affected. Thus, findings in the mice could potentially be attributed to the collection of both plasma and urine under low stress conditions and/or during the daytime, when activity and HR are not as elevated (this study) (Keller et al., 2004). Additionally, because NET is a more prominent mechanism for the removal of NE released in heart compared with other sympathetic targets (Goldstein et al., 1988), collection of whole body blood might not detect increased cardiac NE that is responsible for the observed tachycardia. Plasma DA and EPI were also increased in *NET*^P/P^ mice. A similar effect is observed in NET knockout mice (Keller et al., 2004). Plasma DA probably originates from neuronal release with NE and loss of NET would also prevent reuptake of DA (Goldstein and Holmes, 2008). Excessive EPI might derive similarly from the lack of reuptake, or might be a result of increased EPI release from the adrenal gland. Differential sources of DA and EPI in urine versus plasma could explain the observed selective increases in plasma (Goldstein et al., 2003).

Our results indicate that heart NE content was unchanged in *NET*^+/P^ mice, although it was decreased in *NET*^P/P^ mice in the brain. The fact that heart NE content was largely unaffected by the loss of reuptake and its contribution to NE vesicular stores suggests that neuronal NE synthesis rates are sufficient to compensate for this loss, or that synthesis is upregulated to maintain stores in A457P mice. In support of this possibility, the activity of tyrosine hydroxylase, the rate-limiting enzyme in NE synthesis, is enhanced in both DA transporter and NET knockout mice to maintain appropriate monoamine release levels (Jones et al., 1998; Xu et al., 2000). Interestingly, we show that NET A457P results in reduced heart EPI content, which is believed to rely mainly upon uptake rather than local synthesis (Goldstein et al., 2003). It will be important to determine whether changes in NE synthesis rates in A457P mice account for the maintenance of NE stores despite loss of reuptake.

Although A457P was identified in a cardiovascular syndrome, A457P mice also demonstrate behaviors that are associated with anxiety in both EPM and open field exploration. Our findings are in good agreement with evidence that noradrenergic activity is anxiogenic and that adrenergic receptor antagonists can reverse anxiety behavior (Goddard et al., 2010; Katayama et al., 2010; Kukolja et al., 2008; Morilak et al., 2005; Schank et al., 2008). Indeed, NET knockout mice exhibit increased conditioned freezing (Keller et al., 2006). We did not observe changes in locomotor activity in either the open field or EPM, supporting that the observed behavioral changes reflect increased anxiety (Braun et al., 2011). Our results demonstrate decreased immobility in the TST, consistent with results in NET knockout mice (Xu et al., 2000).

Comorbidity of cognitive and mood disorders with cardiovascular disease is a serious and prevalent problem that is not well understood. Patients with anxiety complain of heart palpitations and patients with tachycardia disorders have a higher incidence of anxiety (Fleet et al., 2003; McCrank et al., 1998). Activation of the SNS has been associated with depression and patients with major depression are at an increased risk for heart disease (Musselman et al., 1998). Interestingly, a cardiac NE uptake deficiency, characterized by decreased clearance of NE in the heart, and decreased DHPG:NE ratios, like that seen in POTS, has been described in individuals with depression (Barton et al., 2007). Individuals with POTS experience increased ratings of depression, anxiety and inattention/ADHD symptoms (Raj et al., 2009). These findings indicate that some comorbidity of cardiovascular and psychiatric disease might involve common underlying genetic determinants, such as NET.

Although a role for CNS NE in stress and anxiety is well established and we predict that central NET dysfunction contributes to anxiety phenotypes in A457P mice, there might also be a cardiovascular influence on brain neurotransmission. Autonomic nervous system input is integrated within brain regions to direct behavioral response to stress, autonomic homeostasis, and emotional and goal-directed behavior. Central noradrenergic neurotransmission is crucial to many of these responses. For example, peripheral cardiovascular and other autonomic stimuli are strong activators of locus coeruleus noradrenergic neuronal firing activity, which in turn drives changes in forebrain activity (Page and Valentino, 1994; Valentino et al., 1991).

Understanding how NET variants can contribute to both cardiovascular disease and CNS dysfunction could have implications for the development of novel treatment strategies. Therapeutics with NET-blocking activity are routinely used to treat mental health disorders (Klimek et al., 1997; Leonard, 1997; Morilak and Frazer, 2004; Ressler and Nemeroff, 1999). Such compounds can have adverse effects on the heart, including drug-induced long QT syndrome (Alvarez and Pahissa, 2010; Vitiello, 2008). Thus, although genetic variation in NET might predispose individuals to disorders indicating the use of NET blockers, such genetic predispositions might also contribute to increased sensitivity to the cardiovascular side effects of medication.

In summary, the present results in NET A457P mice support the contention that NET dysfunction can produce the POTS phenotype as well as behavioral comorbidities reported in POTS (Barton et al., 2007; Fleet et al., 2003; McCrank et al., 1998; Musselman et al., 1998; Raj et al., 2009). Coupled with the emerging evidence for a more widespread incidence of NET deficiency in POTS, our studies argue for our model as an important tool for the assessment of NET-derived plasticities in heart and brain that contribute to patient phenotypes and might provide a path to the development of improved therapeutics.

## MATERIALS AND METHODS

### Generation of NET A457P knock-in mice

Knock-in mice carrying the NET A457P allele (GenBank accession no. AY188506), the orthologous sequence to the human NET A457P allele (GenBank accession no. NM_001043.3), were generated as follows and as shown in Fig. 1. The targeting construct and Southern strategy were generated by Gene Dynamics, LLC. A 5.0 kb 5′ arm and a 5.0 kb 3′ arm were cloned by PCR from 129S6/SvEv genomic DNA. The 5′ arm comprised NET exon and intron sequences from intron 6 through intron 11 and the 3′ arm comprised sequences from exon 11 through the 3′ untranslated region. The A457P mutation was generated in the 5′ arm by site-directed mutagenesis of nucleotide 1387 in exon 9 from a guanosine to a cytosine. The arms were subcloned into a targeting vector, resulting in the placement of a CRE recombinase/neomycin selection cassette flanked by *loxP* sites in intron 11 of *NET*. The CRE was driven by a testis-specific angiotensin converting enzyme promoter to produce excision of the *loxP*-flanked region with germline targeting of the construct. The targeting construct was electroporated into 129S6/SvEv mouse ES cells in the Vanderbilt Transgenic Mouse/ES Cell Shared Resource. Positive ES cell clones were confirmed for homologous recombination by Southern blot and PCR analyses. For Southern blot analysis, ES cell DNA was digested with *Bgl*II, and hybridized with a probe 3′ to and outside of the 3′ arm, with predicted bands of 7.8 and 11.6 kb for the untargeted and recombined *NET* gene, respectively. For PCR of the 3′ end of the targeted vector, a forward primer located within the CRE region and a reverse primer located outside of the 3′ arm (5′-GAGGTCTCTGTGAGGCTGGT-3′ and 5′-CCCCCTCCCAAAATCTAAAG-3′, respectively) were used to generate a 5.1 kb fragment in recombined clones only. Additionally, PCR of the 5′ arm was performed using a forward primer located 5′ to the 5′ arm and a reverse primer located in the 3′ arm (5′-GGGCAGACAGGACTCAGAAG-3′ and 5′-CAGCCCCAGAAGTAGTAGAAGG-3′, respectively), producing a product that includes the A457P site. The PCR products were subjected to dideoxy sequencing for A457P. Chimeras generated from selected ES cells were intercrossed with C57BL6/J mice. Offspring were genotyped using PCR primers spanning the position of the *loxP* site remaining after excision of the floxed region (5′-TGTGTTGGAAGTTCGTGAGC-3′ and 5′-GGCAGAGAGGGAAGAAGGAT-3′) to generate PCR products of 346 and 312 bp for wild-type and targeted mice, respectively. This PCR strategy was used subsequently to genotype all offspring. Additionally, mice in the initial litters were also sequenced for the presence of A457P through PCR and dideoxy sequencing of a region spanning the A457P mutation (5′-CTATAGATGGGAGGCATGGAG-3′ and 5′-CAGCCCCAGAAGTAGTAGAAGG-3′). Speed congenics were used to backcross A457P mice to the C57BL/6 strain in six generations at Jackson Laboratories (Bar Harbor, ME). Speed congenics is a procedure used to decrease the number of generations of breeding required to produce congenic mice on the desired background strain. A panel of single nucleotide polymorphism (SNPs) is genotyped to evaluate the percentage of strain mixture, and breeders for the next generation are selected that contain the highest percentage of the desired strain. This procedure is repeated for approximately six generations to obtain the fully backcrossed mouse line. Data in the present work was generated from fully backcrossed mice as well as earlier generations, and several phenotypes have been replicated in partially and fully backcrossed mice. With the exception of mouse breeding during the speed congenics process, mice were bred and maintained in the Vanderbilt University animal facilities. Mice were housed on 12 hour/12 hour light/dark cycle and given *ad libitum* access to food and water. Mice were greater than 8 weeks old at the start of experiments and were used in accordance with protocols approved by the Vanderbilt University Institutional Animal Care Use Committee.

### Synaptosome preparation

Mice were decapitated, brains dissected on ice, and frontal cortex and hippocampus were dissected and homogenized in 9 ml of ice-cold buffer (0.32 M sucrose, 4.2 mM HEPES, pH 7.4) with a Teflon-glass homogenizer (Wheaton Science Products, Millville, NJ). After centrifugation of the homogenate at 1000 ***g*** for 5 minutes at 4°C, the supernatant was centrifuged at 12,000 ***g*** for 15 minutes at 4°C. Final P2 pellets were resuspended in Krebs-Ringer-HEPES buffer (KRH; 120 mM NaCl, 4.7 mM KCl, 1.2 mM KH_2_PO_4_, 2.2 mM CaCl_2_, 1.0 mM MgSO_4_ and 10 mM HEPES, pH 7.4). Protein concentrations were determined using a bicinchoninic acid (BCA) protein assay (Pierce).

### Radiolabeled transport assay

[^3^H]NE transport was assayed in KRH buffer. Synaptosomes were preincubated for 10 minutes at 37°C, with or without 1 μM desipramine, to assess nonspecific accumulation, followed by the addition of 150 nM (single-point) or varying concentrations of [^3^H]NE (10 nM to 1 μM for kinetic analysis) (∼45 Ci/mmol; PerkinElmer Life Sciences). Transport assays were terminated 10 minutes following the addition of [^3^H]NE by filtration of synaptosomes over 0.3% polyethylenimine-coated glass fiber filters (Whatman GF/B; Whatman) using a cell harvester (Brandel Inc., Gaithersburg, MD). Accumulated [^3^H]NE was determined by liquid scintillation counting (Beckman LS 6000). Peripheral tissues were finely minced and [^3^H]NE (300 nM or 1 μM) was used for a 30-minute transport assay. Following incubation with [^3^H]NE, tissue minces were washed 3× with KRH buffer and accumulated [^3^H]NE determined by liquid scintillation counting.

### Surface biotinylation

To investigate the level of NET surface expression, biotinylation was performed on intact synaptosomes. Synaptosomes were incubated with cell-impermeant sulfosuccinimidyl-2-(biotinamido) ethyl-1,3-dithiopropionate (sulfo-NHS-SS-biotin; 3.0 mg/ml; Pierce) for 30 minutes at 4°C, washed, quenched with 100 mM glycine, extracted in radioimmunoprecipitation assay (RIPA) buffer (10 mM Tris, 150 mM NaCl, 1 mM EDTA, 0.1% SDS, 1% Triton X-100, 1% sodium deoxycholate, pH 7.4, with 250 μM PMSF, 1 μg/ml aprotinin, 1 μg/ml leupeptin, and 1 μM pepstatin) for 30 minutes at 4°C, and then incubated with a 50% slurry of immobilized streptavidin agarose beads (Pierce) for 30 minutes at room temperature. Beads were washed three times in RIPA buffer, and proteins that were bound to streptavidin beads were eluted in 1× Laemmli sample buffer containing 2-mercaptoethanol. Total NET levels were measured in synaptosomes extracted in RIPA buffer. Samples were separated by 10% SDS-PAGE and immunoblotted as described for western blot analyses.

### Western blots

Proteins were electrophoretically transferred overnight at 4°C to Immobilon-P polyvinylidene fluoride membrane (Millipore). Membranes were incubated in blocking solution (PBS, 2% Tween 20, 5% dry milk), followed by incubation with a monoclonal antibody directed against NET at a dilution of 1:500 (NET05; Mab Technologies, Stone Mountain, GA) and incubation with goat anti-mouse HRP-conjugated secondary antibody at a dilution of 1:5000 (Jackson ImmunoResearch). Antibody visualization was performed using Western Lightning chemiluminescence reagents (PerkinElmer Life Sciences). Quantitation of band density was performed using ImageJ (NIH). Gel analyses were performed in replicates, and data are presented with representative blots and graphs of mean optical density from replicate experiments. No differences were observed between male and female A457P mice for NET expression and male and female results were combined for analysis.

### Radiotelemetry

Mouse blood pressure radiotelemetry [Data Sciences International (DSI)] was performed on adult, male NET A457P knock-in mice. Mice were anesthetized with 2–3% isoflurane, and body temperature was maintained with an isothermal pad (Braintree Scientific, Inc., Braintree, MA). After antiseptic preparation, the left carotid artery was isolated, and the lumen was cut to allow insertion of the transmitter catheter to the point of bifurcation. The transmitter body was placed in a lateral subcutaneous pocket. The skin was sutured and secured with adhesive (Nexaband, Veterinary Products Laboratories). The singly-housed mice were monitored in their home cage. BP and HR were recorded continuously in 1-second intervals using flatbed radiofrequency receivers (PhysioTel Receiver RPC-1; DSI) and a digital acquisition system (Dataquest A.R.T., DSI). Data were analyzed by customized MatLab software (Mathworks) in 30-minute increments, averaged over 24-hour time periods or 12-hour time periods, to coincide with the light/dark cycle. Although data was collected continuously, data presented were collected from mice allowed to recover for at least 10 days following surgery.

### ECG recording from anesthetized mice

Under isoflurane anesthesia, mice were instrumented with arterial and jugular vein catheters for electrocardiogram (ECG) recording of HR and BP, and intravenous drug delivery, respectively. Mice were maintained on an isothermal pad throughout the experiment to maintain body temperature at 36–37°C. The arterial catheter was Micro-Renathane tubing [0.025-inch internal diameter (ID); 0.040-inch outer diameter (OD); Braintree Scientific) stretched over hot air to give a tip 300–500 μm in diameter. This tubing was coupled with a smoothed segment of 23 G needle to a 25-cm piece of polyethylene tubing (PE−50; 0.023-inch ID, 0.038-inch OD; Clay-Adams). A small incision was made in the femoral artery and the catheter was advanced into the vessel so that the tip was 0.5 cm above the aortic bifurcation, within the abdominal aorta. Next, an incision was made in the right jugular vein, and a 4- to 5-cm length of Micro-Renathane tubing (0.12-inch ID; 0.25-inch OD) prefilled with heparinized saline (1:1) was inserted and surgically secured. The pharmacological protocol began at the completion of surgery with the mouse remaining under continuous anesthesia. To determine the baroreceptor-mediated cardioinhibitory response, mice underwent a challenge with phenylephrine (PE; 5–40 μg/kg body weight) or nitroprusside (10–40 μg/kg) given intravenously using a syringe pump (CMA 400 pump, CMA Microdialysis, Stockholm, Sweden) in a dose-response manner. Baseline ECG and BP were recorded for 1 minute before drug administration, and the post-drug response was recorded for 3 minutes after administration. The ratio of the maximal change in HR over the change of mean systolic BP was calculated and averaged at each dose in each animal. BRS was determined by the averaged ratio of HR change [change in the R-wave to R-wave interval (RRI) over systolic BP (SBP) change (ΔRRI/ΔSBP)].

### Catecholamine assay

Catecholamines and metabolites were measured in plasma, urine and tissue. Blood was collected via cardiac puncture in isoflurane-anesthetized mice and preserved with EGTA and reduced glutathione, and organs were harvested immediately thereafter. Spot urine was collected at ∼9:00 am from awake, unstressed mice placed in individual weighboats. 6 N HCl was added to urine samples to prevent catechol breakdown. Urine and plasma were alumina extracted and tissues were extracted in 0.1 N perchloric acid. Catecholamine and metabolite concentrations were determined by high-performance liquid chromatography combined with electrochemical detection (HPLC-EC). 3,4-dihydroxybenzylamine (DHBA) was used as an internal standard in all samples. Urine catecholamine and metabolite measures were normalized to urine creatinine levels.

### Behavior

#### Open field activity

All behavior was performed in the Vanderbilt Murine Neurobehavioral Laboratory. For the open field test, mice were placed in open field activity chambers (29×29×20.5 cm) that were housed inside environmental chambers (Med Associates, Inc., St Albans, VT). The environmental chambers remained closed for the duration of the test. The testing room was kept in normal lighting (250–300 lux) during testing. Testing was begun at ∼9:00 am. Mice were exposed to the open field for 1 hour. Movement and location were detected by the interruption of infrared photoelectric beams by the mouse and distance travelled in center and surrounding zones was calculated in 5-minute blocks (Med Associates).

#### Elevated plus maze

The elevated plus maze (EPM) consisted of two closed and two open arms connected by a center zone (dimensions are 67 cm length×6.5 cm width×15 cm height in the closed arms). The EPM was set on a table during testing. The testing room was kept in normal lighting (250–300 lux). Testing was begun at ∼9 am. Mice were brought into the testing room, in home cages, 30 minutes prior to testing with free access to food and water. To initiate the test, mice were lowered by the tail onto the center of the maze, directly next to and facing a closed arm. Test duration was 5 minutes and was video-recorded by an overhead camera. Time spent in each zone, number of entries into each zone and freezing behavior were analyzed using ANY-maze software (Stoelting, Wood Dale, IL). Head dips were counted by an observer blind to mouse genotype.

#### Tail suspension

Mice were brought into an anteroom 30 minutes prior to testing. For the tail suspension test, mice were suspended for 7 minutes by the tail from a vertical aluminum bar (bar size: 11.5×2.3 cm) attached to the top of a box-like enclosure (33×33×32 cm) open on the front side (Med Associates). Mice were attached to the bar by tape placed ∼1.5 cm from the tip of the tail. Mice were monitored visually throughout the test to ensure they did not tail climb. The testing room was kept in normal lighting (250–300 lux) during the test. Testing was begun at ∼9:00 am. The level and duration of force placed on the bar by the mouse was measured, with a force level below the lower threshold being counted as immobilization (Med Associates). The following settings were used: lower threshold of 7, upper threshold of 20, gain of 8, and resolution of 220 milliseconds.

### Statistics

Data were analyzed by one- or two-way ANOVA followed by post-hoc testing, or by two-tailed *t*-tests, as appropriate for experimental design and *a priori* predictions (SPSS20, IBM; Prism version 5, GraphPad Software Inc.). *P*-values <0.05 were considered significant. Single point transport and western blot assays were analyzed by two-tailed *t*-tests for each brain region. Transport experiments to determine saturation kinetics were analyzed using nonlinear regression analysis according to a single-site hyperbolic model to calculate *K*_M_ and *V*_MAX_ values (Prism version 5; GraphPad Software Inc.). One-way ANOVA was used to compare derived *V*_MAX_ or *K*_M_ values. Plasma and urine neurochemistry was analyzed by two-way ANOVA followed by post-hoc tests. Telemetry data were analyzed by two-way ANOVA followed by post-hoc tests.
